# Retraction: Induction of heat shock protein expression in SP2/0 transgenic cells and its effect on the production of monoclonal antibodies

**DOI:** 10.1371/journal.pone.0343942

**Published:** 2026-03-03

**Authors:** The PLOS One Editors

After this article [1] was published, concerns were raised regarding the statistical analyses and interpretation of the results. Specifically:

- Figs 1A, 2, 3A, 5 and 7A show statistical significance markers for all conditions including for the control group, despite the control group acting as the comparator for the other treatment conditions.
- The Statistical analysis subsection of the Materials and methods section and the description of the statistical tests in the figure legends lack sufficient detail for replication.
- The article presents statements and conclusions that are not supported by the results, including:
  - The expression of HSPs was greater in paracetamol-treated cells than in heat shock-treated cells.
  - Cells treated with paracetamol at concentrations above 0.5 mM showed a significant decrease in cell viability after 24 hours.
  - Heat shock treatment decreased cell viability after 24 hours.

The corresponding author stated that they agreed with the above concerns for the statistical analyses. During post-publication correspondence, they provided multiple statistical re-analyses for all graphs in [1]. A member of the *PLOS One* Editorial Board reviewed the author’s responses and repeated statistical analyses, and stated that these did not resolve the concerns raised for the statistical methods and that the re-analyses raised additional concerns for the reliability of the results reported.

Following editorial assessment, the *PLOS One* Editors concluded that the article does not meet the journal’s third and fourth criteria for publication [2]. In light of the above concerns, which call into question the validity and reliability of the published results, the *PLOS One* Editors retract this article.

All authors agreed with the retraction and apologize for the issues with the published article.
